# Not spreading in reverse: The dewetting of a liquid film into a single drop

**DOI:** 10.1126/sciadv.1600183

**Published:** 2016-09-28

**Authors:** Andrew M. J. Edwards, Rodrigo Ledesma-Aguilar, Michael I. Newton, Carl V. Brown, Glen McHale

**Affiliations:** 1 School of Science and Technology, Nottingham Trent University, Clifton Lane, Nottingham NG11 8NS, U.K.; 2 Smart Materials and Surfaces Laboratory, Department of Physics and Electrical Engineering, Northumbria University, Ellison Place, Newcastle upon Tyne NE1 8ST, U.K.

**Keywords:** Dewetting, dielectrowetting, contact-line dynamics, slip

## Abstract

Wetting and dewetting are both fundamental modes of motion of liquids on solid surfaces. They are critically important for processes in biology, chemistry, and engineering, such as drying, coating, and lubrication. However, recent progress in wetting, which has led to new fields such as superhydrophobicity and liquid marbles, has not been matched by dewetting. A significant problem has been the inability to study the model system of a uniform film dewetting from a nonwetting surface to a single macroscopic droplet—a barrier that does not exist for the reverse wetting process of a droplet spreading into a film. We report the dewetting of a dielectrophoresis-induced film into a single equilibrium droplet. The emergent picture of the full dewetting dynamics is of an initial regime, where a liquid rim recedes at constant speed and constant dynamic contact angle, followed by a relatively short exponential relaxation of a spherical cap shape. This sharply contrasts with the reverse wetting process, where a spreading droplet follows a smooth sequence of spherical cap shapes. Complementary numerical simulations and a hydrodynamic model reveal a local dewetting mechanism driven by the equilibrium contact angle, where contact line slip dominates the dewetting dynamics. Our conclusions can be used to understand a wide variety of processes involving liquid dewetting, such as drop rebound, condensation, and evaporation. In overcoming the barrier to studying single film-to-droplet dewetting, our results provide new approaches to fluid manipulation and uses of dewetting, such as inducing films of prescribed initial shapes and slip-controlled liquid retraction.

## Teaser

Dewetting films are not the time reversal of spreading droplets.

## INTRODUCTION

It is a familiar observation that a water film covering a glass surface will spontaneously break into puddles, and small enough puddles will bead up to form spherical cap–shaped droplets (*1*–*3*). These dewetting processes occur when a thin liquid film is energetically unfavorable relative to the puddle/droplet configuration, and are remarkably commonplace. In some situations, such as drying and cleaning, the spontaneous and fast dewetting of liquids is essential to obtain smooth, impurity-free surfaces (*4*, *5*). In others, such as coating and lubrication, a primary aim is to avoid dewetting or to at least retard it (*6*, *7*). Therefore, there is a strong interest among the academic and industrial communities in understanding and harnessing the dewetting of liquids from solid surfaces (*8*).

The details of how a liquid front transitions from a film to a drop shape have important implications, both for fundamental and practical applications. For instance, the ability to predict and control the speed of dewetting is inherently linked to the dynamics of the triple line (*1*) and is critical for developing smart self-cleaning surfaces (*9*–*11*) and more robust coating agents (*12*).

Since the pioneering work of Redon *et al*. (*13*), substantial effort has been made to understand the stability and dewetting dynamics of thin films. The vast majority of studies focusing on the dynamics of the retracting film track the growth of dry areas on solid surfaces (*14*–*16*). These are usually obtained by bursting the liquid film to form “holes,” which eventually reconnect to form puddles and drops. Gravity aside, the dewetting dynamics can be thought of as the transition from a film state to a final drop state. Despite the apparent simplicity of the problem, direct measurements of the liquid dynamics over the whole transition have remained elusive.

At first sight, the dewetting of a film seems to be the counterpart of the spreading of a droplet. Spreading involves the replacement of a solid-gas interface by a solid-liquid interface, whereas the reverse is true for dewetting. Moreover, in both cases, the initial configuration is an out-of-equilibrium state, which relaxes to a final equilibrium state where the two states are a film and a droplet, respectively. Therefore, one might imagine that the slow dynamics associated with the relaxation of a liquid film could be described as a simple time reversal of the spreading problem. Specifically, because a small droplet spreading into a film goes through a smooth sequence of spherical cap shapes before approaching a film state, one might anticipate that a dewetting film goes through a smooth sequence of spherical cap shapes until it achieves its final equilibrium droplet shape. However, studies of dewetting via the growth of dry holes from a film only provide indirect evidence, and so, the details of the dewetting process are an open question. A significant difficulty in directly observing the time-reversed process of a film dewetting into a droplet is the inability to form a well-defined initial liquid film shape on a surface for which the equilibrium state is a spherical cap droplet. Recently, we have developed a technique on the basis of dielectrowetting, which allows us to induce superspreading using a voltage, so that a liquid can be forced to spread into a thin film on a nonwetting substrate (*17*). This opens up a new possibility of looking at the relaxation of the liquid from a film state to a droplet state by removing the dielectrowetting voltage acting on a thin liquid film to effectively instantaneously quench the state of the system, leaving it subject to only interfacial forces. Moreover, by designing specifically shaped electrodes, a range of prescribed initial film shapes, such as stars and arrays of distinct features, could be created and the process of their dewetting could be studied.

## RESULTS

### Dielectrowetting-induced thin liquid films

**Fig. 1:**
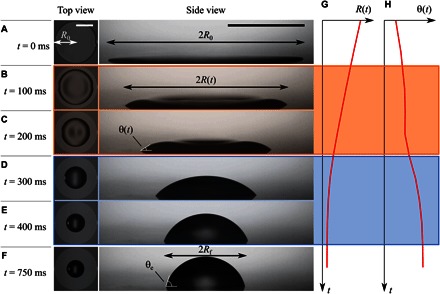
Experimental imaging of the dewetting of a liquid droplet from a smooth solid surface. (**A**) A liquid droplet (TMPTGE; Ω = 1.45 μl, μ = 180 mPa s, γ = 43 mN m^−1^, *T* = 22°C, θ_e_ = 70°) is forced to wet a circular Teflon patch using a dielectrowetting setup. The resulting pancake-shaped liquid film, which corresponds to the initial configuration of the experiment, has a radius *R*_0_ = 2.5 mm. At time *t* = 0 ms, the dielectrowetting voltage is removed. The dewetting dynamics that follows is tracked by recording the instantaneous film radius, *R*(*t*), and the apparent contact angle averaged between the left and right film edges, θ(*t*). Scale bars, 2 mm. (**B** and **C**) At intermediate times, an annular rim forms close to the contact line. The rim is visible from both the top and side views of the film. (**D** and **E**) At long times, the rim merges and the film relaxes to a spherical cap shape. (**F**) Equilibrium state of the droplet, where the radius and contact angle reach constant values, *R*_f_ and θ_e_. (**G** and **H**) Representative curves for the base radius and apparent contact angle. The formation of the rim correlates with a linear decrease in the radius and a plateau in the contact angle. The merging of the rim into a spherical cap gives way to a relaxation stage where the radius and contact angle relax to their final equilibrium values.

In our experiments, which are summarized in Fig. 1 (see also movies S1 and S2), we recorded the evolution of thin films of the isotropic liquid trimethylolpropane triglycidyl ether (TMPTGE; Chemical Abstracts Service no. 3454-29-3) deposited on a smooth substrate. The substrate consists of a set of alternating interdigitated electrodes (IDEs) arranged to form a circular surface covered by a thin insulating layer and Teflon surface treatment (see Materials and Methods for details of the electrode geometry). In the presence of a voltage *V*, the electrodes generate a nonuniform electric field, which results in a liquid dielectrophoresis energy proportional to *V*^2^ in addition to the surface energies of the gas-liquid, solid-liquid, and gas-solid interfaces. The liquid responds by spreading on the solid until it covers an area that satisfies the overall energy balance. By tuning the liquid volume, Ω ≈ 1 μl, and the electrode voltage, *V* ≈ 400 V, we can control the spreading of the liquid. This results in axisymmetric “pancake”-shaped liquid films that cover a circular surface area up to the base radius of the electrode patch, *R*_0_ = 2.5 mm, and thus have a thickness of *h*_0_ ≈ Ω/π*R*_0_^2^ ~ 50 μm (Fig. 1A).

Once the initial spreading of the droplet into a thin film was achieved, we suddenly removed the applied voltage. Such a quench leads to an abrupt change in the energy balance, which then results in a new equilibrium state purely determined by the interfacial energies, thus driving the dewetting of the liquid film. Using high-speed imaging, we tracked the liquid-air interface dynamics during the dewetting process. Contrary to the slow, quasi-static dynamics of a spreading drop, a dewetting film exhibits a very different pathway toward its equilibrium configuration. Rather than a spherical cap, we observe the formation of a receding rim spanning the circumference of the film (Fig. 1, B and C). It is only at long times that the rim relaxes to a spherical cap shape (Fig. 1, D and E) and finally reaches the equilibrium configuration (Fig. 1E). This is different from the time reversal of a droplet spreading to a film, which would show a sequence of shapes shown in Fig. 1 (A and D to F) without the intermediate shapes shown in Fig. 1 (B and C).

Therefore, the dewetting process observed here has two regimes: an initial regime (Fig. 1, A to C) displaying a rim and a central dimple followed by a subsequent spherical cap droplet retraction regime. To quantify both regimes, we measured the time dependence of the base radius of the liquid, *R*(*t*), and the apparent contact angle, θ(*t*), which was determined by fitting a third-degree polynomial to the side-view projection of the liquid-gas interface and extrapolating a tangent to the intersection with the solid (see Materials and Methods for further details). Figure 1G shows a clear correlation between the presence of the rim with a linear decrease in the radius with time, indicating that the dewetting speed, d*R*/d*t* ~ 1 mm s^−1^, is constant. At long times, we observe a crossover to a relaxation regime, which corresponds to the merging of the rim to form a spherical cap. In this regime, the contact line slows down as it approaches its final equilibrium position. Figure 1H shows the time evolution of the contact angle, which has a nonmonotonic relaxation toward equilibrium. At short times, corresponding to the formation of the rim, θ rapidly reaches a plateau, where it remains approximately constant. At longer times, when the rim gives way to the formation of the spherical cap, there is a crossover toward the final relaxation, where θ reaches its equilibrium (Young’s) contact angle, θ_e_ ≈ 70°.

At the recorded temperature of the experiment, *T* = 22°C, TMPTGE has a surface tension γ = 43 mN m^−1^, dynamic viscosity μ = 180 mPa s, and mass density ρ = 1157 kg m^−3^. This gives a Reynolds number, *Re* = ρ|d*R*/d*t*|*R*/μ ~ 10^−3^, and a Weber number, *We* = ρ|d*R*/d*t*|^2^*R*/γ ~ 10^−5^, thus ruling out the effects of inertia relative to both viscous and capillary forces. The capillary length, *l*_c_ = (γ/ρ*g*)^1/2^ ≈ 2 mm, is larger than the final height of the droplets, *H*_f_ ≈ 1 mm. This implies that any effects due to gravity are negligible. The capillary number, *Ca* = μ|d*R*/d*t*|/γ, is of order 10^−2^, indicating that surface tension dominates over viscous bending, something that appears to contradict the strongly distorted interface shapes observed in the experiments.

**Fig. 2:**
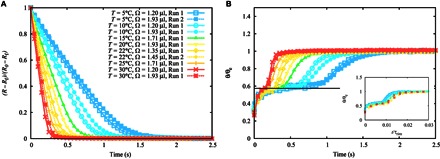
Base radius and apparent angle as a function of time during the dewetting of liquid films. (**A**) At intermediate times, the base radius decreases linearly in time, with a dewetting speed that increases with increasing temperature and decreasing volume. (**B**) The apparent contact angle, θ, is normalized using the equilibrium contact angle, θ_e_. The linear dewetting regime, where the speed of the contact line is constant, corresponds to the first plateau. At longer times, there is a crossover to a second plateau, corresponding to the equilibrium state of the drop. The data collapse to a single master curve upon rescaling time by the relaxation time of the rim, τ_rim_. The solid line corresponds to the theoretical prediction for the first plateau (see text).

To test the robustness of our first observations, we conducted a series of experiments varying the temperature of the experimental chamber in the range 5°C < *T* < 30°C. Increasing the temperature has the effect of decreasing the viscosity of TMPTGE with a weak decrease in the liquid-air surface tension (see Table 1 for a summary of experimental conditions and parameters). By tracking the base radius and the apparent contact angle of the film as a function of time, we confirmed the two distinct dewetting regimes (see Fig. 2). Because the volume of the liquid has small variations between different experiments, drops reach slightly different final base radii. In addition, the weak dependence of surface tension on temperature in our liquid has a small effect on the equilibrium contact angle. Therefore, it is convenient to rescale the raw data using the dimensionless variables (*R* − *R*_f_)/(*R*_0_ − *R*_f_) and θ/θ_e_. Figure 2A shows a plot of the rescaled base radius as a function of time and reveals that the dewetting proceeds faster with increasing temperature and decreasing droplet volume. Curves of the apparent contact angle are shown in Fig. 2B and show that the contact angle always reaches the same transient plateau, whose duration extends for a longer time at lower temperatures corresponding to higher viscosities, before crossing over to the final relaxation toward equilibrium.

**Table 1:** Summary of experimental conditions. Errors for the volume, surface tension, and viscosity correspond to the accuracy of the measuring apparatus. The error in the contact angle is the SD of the sample.

**Handle**	***n***	**Ω (μl)**	***T*** **(°C)**	**γ (mN m^−1^)**	**μ (mPa s)**	**θ_e_ (°)**	**Symbol**
D94 5	2	1.20 ± 0.03	5	44.78 ± 0.25	686 ± 20	71 ± 1	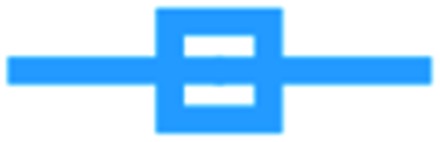
D96 5	1	1.93 ± 0.03	5	44.78 ± 0.25	686 ± 20	71	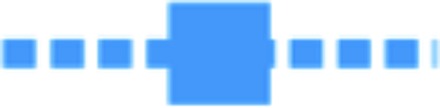
D94 10	2	1.20 ± 0.03	10	44.28 ± 0.25	437 ± 20	71 ± 1	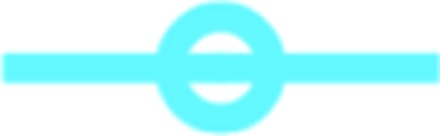
D96 10	2	1.93 ± 0.03	10	44.28 ± 0.25	437 ± 20	71 ± 1	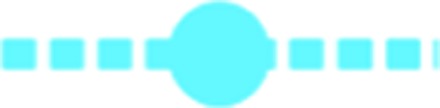
D97 15	4	1.71 ± 0.03	15	43.77 ± 0.25	299 ± 20	72 ± 1	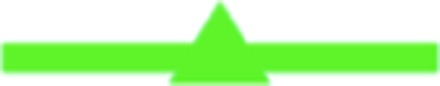
D96 20	2	1.93 ± 0.03	20	43.27 ± 0.25	210 ± 20	71 ± 1	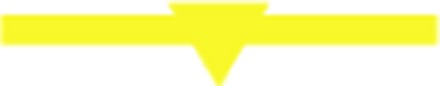
D89 22	5	1.35 ± 0.03	22	43.06 ± 0.25	180 ± 20	73 ± 2	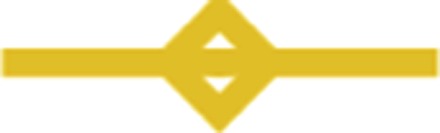
D92 22	7	1.45 ± 0.03	22	43.06 ± 0.25	180 ± 20	75 ± 2	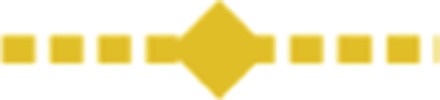
D97 25	4	1.71 ± 0.03	25	42.76 ± 0.25	149 ± 20	75 ± 2	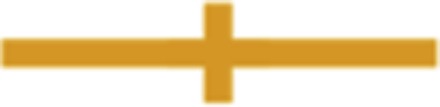
D94 30	2	1.20 ± 0.03	30	42.25 ± 0.25	109 ± 20	74 ± 1	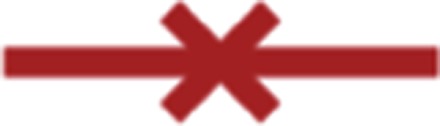
D96 30	2	1.93 ± 0.03	30	42.25 ± 0.25	109 ± 20	72 ± 1	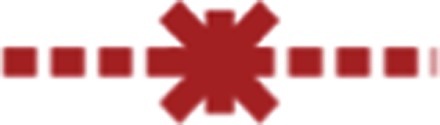

### Lattice Boltzmann simulations of a dewetting film

The observation of the two dewetting regimes is intriguing, particularly regarding the selection of the dewetting speed and contact angle at intermediate times. To gain a better insight into the liquid dynamics, we conducted a series of lattice Boltzmann numerical simulations of a Newtonian liquid film dewetting from a flat solid surface (see Materials and Methods for a presentation of the model equations and the numerical algorithm) (*18*). Because the experimental film shapes are axisymmetric, we carried out two-dimensional simulations. Furthermore, from the experiments, the dominant effect of temperature is expected to be a change in the surface tension and liquid viscosity, and thus, the simulations were focused on tracking the dynamics of dewetting films at different values of these parameters while keeping the Reynolds, *Re*, Weber, *We*, and capillary numbers, *Ca*, to small values.

**Fig. 3:**
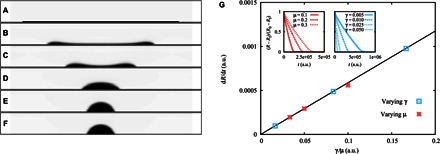
Lattice Boltzmann simulations of a dewetting film. (**A** to **F**) Instantaneous liquid profiles relaxing from an initial film configuration of aspect ratio *h*_0_/*R*_0_ = 0.02 on a surface where the equilibrium contact angle is θ_e_ = 70°. In agreement with experiments, the droplet forms a rim (B and C), which eventually merges to form a spherical cap (D and F). (**G**) The speed of the dewetting rim increases with increasing surface tension and decreasing viscosity (insets). a.u., arbitrary units. Simulation data collapse onto the same scaling curve d*R*/d*t* ~ γ/μ.

In our experiments, the initial pancake-shaped films can be approximately described by a slab geometry with an aspect ratio *h*_0_/*R*_0_ ≈ 0.02. Figure 3 (A to F) shows a simulation time sequence for such an initial condition, which is allowed to relax on a surface where the equilibrium contact angle is θ_e_ = 70°. Figure 3 (A to C) shows the formation of a rim, which eventually merges to form a spherical cap as shown in Fig. 3 (D to F), and thus supports the idea that the rim formation is a generic feature of liquid dewetting films and not specific to the liquid used in the experiments.

The smallness of *Re*, *We*, and *Ca* observed in the experiments suggests that the liquid dynamics is controlled by surface tension and viscous friction. Following previous results of wetting dynamics (*1*), the contact line speed is expected to scale with the capillary speed *U*_Ca_ = γθ_e_^3^/9μ. Our simulation results, shown in Fig. 3G, show that the velocity of the rim (measured from the linear section of the inset curves) has the scaling d*R*/d*t* ~ γ/μ, and thus confirm the effect of viscosity and surface tension on the rim dynamics.

**Fig. 4:**
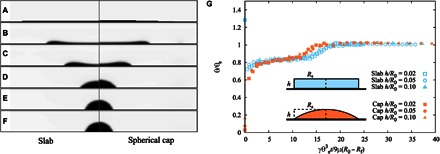
Lattice Boltzmann simulations of dewetting films of different initial shapes. (**A** to **F**) Instantaneous liquid profiles relaxing from slab (left) and spherical cap (right) initial configurations. In both cases, the aspect ratio of the initial liquid shape is set to *h*_0_/*R*_0_ = 0.02, and the final equilibrium contact angle is θ_e_ = 70°. (**G**) Apparent contact angle as a function of time for different initial aspect ratios of slab and spherical cap–shaped films. The time axis is rescaled by the time scale τ_rim_ = 9μ(*R*_0_ − *R*_f_)/γθ_e_^3^. The good collapse of the data shows that the rim speed scales with the capillary speed, *U*_Ca_ = γθ_e_^3^/9μ, and that the duration of the linear dewetting regime scales with the amplitude of the lateral distortion, *R*_0_ − *R*_f_.

Intuitively, the time scale of retraction of the rim should depend on the initial film shape. To explore this effect, we carried out simulations focusing on the effect of the initial height-to-radius aspect ratio *h*_0_/*R*_0_ while keeping the volume, viscosity, surface tension, and equilibrium contact angle to fixed values. To ensure that our deductions do not depend on the details of the initial shape of the film, we carried out simulations comparing the relaxation of the liquid with either initial spherical cap or slab shapes toward the same equilibrium state. Figure 4 (A to F) shows a time lapse of simulation snapshots of the relaxation of an initial slab-shaped film (left-hand panels) compared to a liquid of the same volume starting from a spherical cap configuration (right-hand panels). In both cases, the aspect ratio between the maximum height and the initial base radius of the liquid was set to *h*_0_/*R*_0_ = 0.02. As shown in the left panels of Fig. 4 (A to F), the initial shape is quickly lost, leading to the development of the rim, which is therefore robust against changes in the initial shape of the liquid and is conserved even when the initial shape of the film corresponds to a spherical cap. Simulations for different initial aspect ratios of the film show that, for a given initial shape (cap or slab), the retraction of the rim extends for longer times at smaller *h*_0_/*R*_0_. This is expected, because films are more distorted from their equilibrium shape at small aspect ratios. These observations suggest that the typical relaxation time of the rim scales as τ_rim_ ~ (*R*_f_ − *R*_0_)/*U*_Ca_. In Fig. 4G, we present simulation results for the apparent contact angle as a function of time for different initial values of the aspect ratio *h*_0_/*R*_0_, for both slab and spherical cap configurations. When rescaling time using τ_rim_, the data show a good collapse over the region where the contact angle remains constant, regardless of the initial shape of the film. Using the same scaling for time gives an excellent collapse of the experimental data onto a master curve during the rim retraction, as shown in the inset of Fig. 2B.

These observations support the idea that, unlike spreading, dewetting cannot be described as a smooth succession of spherical cap shapes toward the final equilibrium shape. Upon closer inspection, the interface profiles in our simulations indicate a local mechanism driving the dewetting of the front. At short times, when *t* << τ_rim_, the interaction with the solid induces a sudden change in the apparent contact angle (Fig. 4, A and B) (*19*). The interface thus develops a nonuniform curvature close to the contact line and a corresponding rise in the capillary pressure. The front evolves to smooth out the curvature gradient subject to the boundary condition imposed by the apparent contact angle. The result is the accumulation of liquid close to the contact line in the form of a smooth rim at times comparable to τ_rim_ (Fig. 4, B and C). Because the curvature of the rim is in excess of the curvature of the equilibrium spherical cap, the rim recedes with an apparent contact angle that is smaller than the equilibrium contact angle. During this stage, the curvature of the front slowly decreases as the rim gathers more fluid. The corresponding balance between an approximately constant driving capillary force and viscous friction thus suggests a constant dewetting speed and a constant dewetting angle, as observed in both our experiments and numerical simulations. At long times relative to τ_rim_, the rim merges at the center of the drop, and the smoothing out of the remaining excess curvature gives rise to the final relaxation to an equilibrium spherical cap configuration, with the corresponding slowing down of the contact line and final relaxation of the apparent angle to the equilibrium contact angle (Fig. 4, D to F).

### Hydrodynamics of a dewetting film

**Fig. 5:**
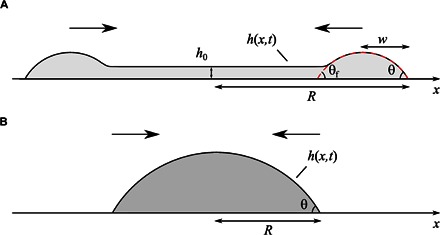
The two dynamic dewetting regimes of a liquid film. (**A**) Schematic shape of the cross-sectional profile of a dewetting film. The cross section of the rim corresponds to two independent structures of width *w*, connected by a thin film of thickness *h*_0_. The shape of the rim is described by the apparent contact angles θ and θ_f_. (**B**) Schematic of the quasi-equilibrium shape of a dewetting droplet.

**Governing equations.** On the basis of our previous observations, we are now in a position to analyze the experimental results in the context of a continuum hydrodynamic model. The key governing equation describing the dynamics of the interface in the limit of negligible inertia (zero *Re* and *We*) and small capillary number, *Ca*, is the thin-film equation (*1*, *8*)

$$\frac{\partial h}{\partial t}=\frac{\gamma }{3\mu }\frac{\partial }{\partial x}(h^{2}(h+3\lambda )\frac{\partial^{3}h}{\partial x^{3}})$$ (1)

which describes the evolution of a gently curved interface profile, *h*(*x*,*t*), intersecting the solid at a distance *x* = *R*(*t*) from the origin, that is, *h*(*R*,*t*) = 0 (see Fig. 5). To account for the motion of the contact line, the model allows a finite slip velocity at the solid wall (located at *h* = 0), controlled by the so-called Navier slip length, λ (*20*).

**Dewetting at intermediate times.** We first focus our attention on the relaxation of the film at intermediate times. From our observations, the shape of the interface corresponds to a rim connected to a thin film of thickness *h*_0_ (Fig. 5A). The speed of the moving contact line is set by the balance between the rate of work done by capillary forces, d*W*_cap_/d*t*, and the viscous energy dissipation, *T*d*S*/d*t*, caused by the flow pattern in the liquid (*13*). Within the lubrication approximation, the capillary work per unit length of the contact line scales as d*W*_cap_/d*t* ~ γ(θ^2^ − θ_e_^2^)d*R/*d*t*, whereas the viscous dissipation obeys *T*d*S*/d*t* ~ μln(*w*/λ)(d*R/*d*t*)^2^/θ, where *w* is the width of the rim. On the basis of these scalings, one expects d*R/*d*t* ~ γθ_e_^3^/μln(*w*/λ).

To quantify these ideas in the context of our experiments, we follow the analysis of Eq. 1 carried out by Snoeijer and Eggers (*21*), which works out the hydrodynamics of a dewetting film in detail and provides a theoretical understanding of the dewetting process at intermediate times. Using the method of asymptotic expansions, they matched the shape of the rim to the thin film lying behind it. Close to the contact line, the driving capillary force is balanced by viscous stresses extending from the liquid wedge, where the thickness of the rim is comparable to the slip length λ up to the width of the rim, *w*. The apparent contact angle of this portion of the rim, θ, obeys the relation

$$\theta^{3}={\theta_{e}}^{3}-9Ca\;\text{ln}(\frac{2\theta_{e}w}{3\lambda })$$ (2)

On the other hand, the rim connects smoothly to the film lying behind it. The apparent contact angle in this portion of the rim is

$${\theta_{f}}^{3}=9Ca\;\text{ln}(\frac{2aCa^{1/3}w}{eh_{0}})$$ (3)

where the constant *a* = 1.094… is a result of the matching approximation and *e* = exp(1). The smallness of *Ca* thus implies that the rim retains a gently curved shape, that is, θ_f_ ≈ θ. Imposing this condition to eliminate θ from Eqs. 2 and 3 gives the following relation between the instantaneous contact line speed and the equilibrium contact angle

$$\frac{dR}{dt}=-\frac{\gamma {\theta_{e}}^{3}}{9\mu }{\left[\text{ln}(\frac{4a}{3e}\theta_{e}Ca^{1/3}\frac{w^{2}}{h_{0}\lambda })\right]}^{-1}$$ (4)

**Fig. 6:**
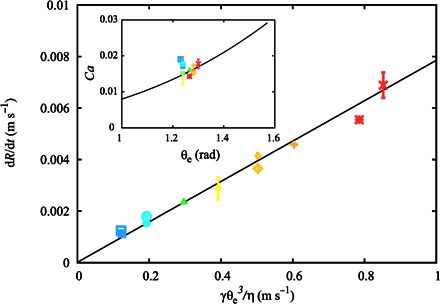
Scaling of the interface speed in the linear regime of a dewetting film. Speed of the receding rim as a function of the capillary speed *U*_Ca_ = γθ_e_^3^/9μ. The solid line is a visual guide. The inset shows a direct comparison of the experimental data (symbols) with the hydrodynamic theory (solid line). Error bars correspond to 1 SD of the sample. Experimental parameters for each symbol are summarized in Table 1.

Equation 4 justifies the proposed scaling d*R*/d*t* ~ *U*_Ca_ = γθ_e_^3^/μ, which we confirm against the experimental data in Fig. 6. Apart from the weak dependence on time through *Ca* and *w*, the logarithmic factor in Eq. 4 encodes the viscous dissipation within the retreating rim. This is controlled by the length scale separation between the width of the rim, *w*, and the relevant microscopic length scale, which in this instance is the slip length, λ. Overall, the logarithmic factor sets the order of magnitude of the contact line speed, d*R*/d*t*, for given values of the surface tension, viscosity, and equilibrium contact angle. In our experiments, *w* ≈ 1 mm and *h*_0_ ≈ 50 μm. We fix the slip length to a value comparable to the molecular size, λ = 1 nm (*22*, *23*). This is a reasonable assumption, because λ enters the theory through a weak logarithmic dependence. Using these values in Eq. 4, we confirm that the order of magnitude of the capillary number is *Ca* ≈ 10^−2^, in agreement with the experimental results (see inset in Fig. 6).

The quantitative agreement between the experiments and the hydrodynamic model explains the apparent contradiction of a rim shape far from the quasi-equilibrium spherical shape, yet at low values of the capillary number. As the rim forms, its shape is determined by the local balance of viscous and capillary forces. The dominant role of capillary pressure ensures a smooth profile. Because the capillary number remains constant in the linear regime, the apparent contact angle also remains constant. The selection of the apparent contact angle is thus a result of the force balance and can be deduced from Eqs. 2 and 3 (*21*)

$$\frac{\theta }{\theta_{e}}={\left[1+\frac{\text{ln}(\frac{2\theta_{e}w}{3\lambda })}{\text{ln}(\frac{2eaCa^{1/3}w}{h_{f}})}\right]}^{-1/3}$$ (5)

Using the parameter values of our experiments, where *Ca* ≈ 10^−2^, we find θ/θ_e_ ≈ 0.58, in close agreement with the plateau observed in Fig. 2.

**Long-time relaxation.** We now turn our attention to the relaxation of the droplet at long times, where it takes the shape of a spherical cap. The balance between viscous bending and capillary forces, encoded in the capillary number, determines the instantaneous apparent contact angle of the spherical cap. For small to moderately large values of the apparent angle (θ < 135°), Eq. 1 gives a similar result to Eq. 2, often called the Cox-Voinov relation (*1*)

$$\theta^{3}={\theta_{e}}^{3}-9Ca\;\text{ln}(\frac{R}{2e^{2}\lambda })$$ (6)

As the drop approaches equilibrium, the behavior of the apparent contact angle can be characterized by considering Δθ = θ_e_ − θ, which is the deviation from the equilibrium contact angle. Then, expanding Eq. 6 in powers of Δθ leads to

$$\Delta \theta \approx \frac{3Ca}{{\theta_{e}}^{3}}\text{ln}(\frac{R}{2e^{2}\lambda })$$ (7)

The base radius is related to the volume of the droplet and the contact angle by geometry, and to leading order in Δθ. This leads to the following relation between the speed of the interface and the rate of change of Δθ

$$\frac{dR}{dt}={\left(\frac{3\Omega }{\pi f(\theta_{e})}\right)}^{1/3}\frac{d(\Delta \theta )}{dt}$$ (8)

where $f(\theta_{e})\approx 81\theta_{e}^{4}/4$ for small angles, which remains within 10% of the exact result for the contact angles considered here.

Combining Eqs. 7 and 8 gives a prediction of an exponential approach to equilibrium, Δθ ≈ τd(Δθ)/d*t*, where

$$\tau =\frac{3\mu }{\gamma }{\left(\frac{4\Omega }{\pi {\theta_{e}}^{10}}\right)}^{1/3}\text{ln}(\frac{R_{f}}{2e^{2}\lambda })$$ (9)

is the relaxation time scale of the interface close to equilibrium.

**Fig. 7:**
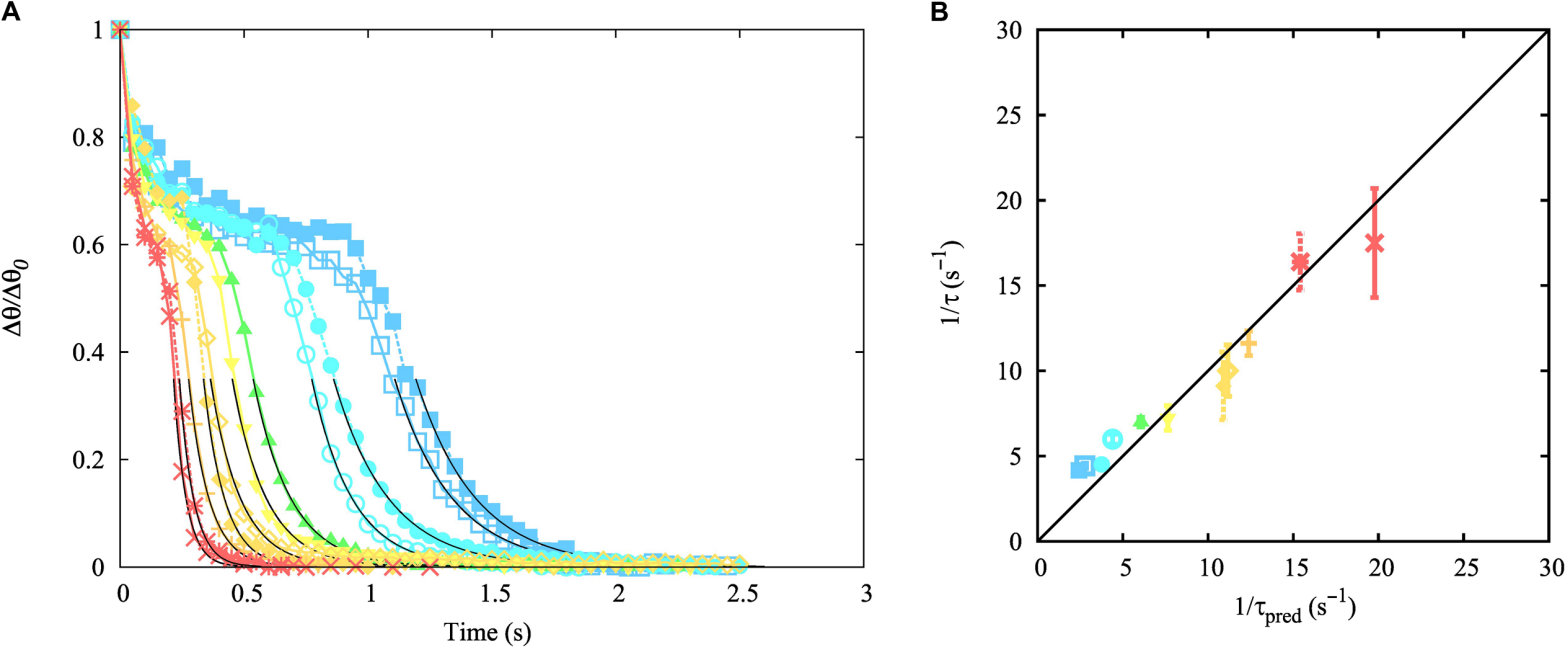
Exponential approach to equilibrium of a dewetting droplet. (**A**) Difference between the equilibrium and apparent contact angles as a function of time. The solid line corresponds to a fitted exponential function. (**B**) Scaling of the relaxation time in the exponential regime. The inverse relaxation time is compared to the theoretical prediction. Error bars correspond to 1 SD of the sample. Experimental parameters for each symbol are summarized in Table 1.

Experimentally, the data show agreement with an exponential approach to equilibrium in the long-time relaxation limit as the final equilibrium droplet state is established. This is confirmed in Fig. 7A, which shows a plot of the deviation from the equilibrium contact angle, Δθ, as a function of time (data for the base radius also follow an exponential slowing toward the final radius, *R*_f_). Quantitatively, the relaxation time constant (or equivalently relaxation rate) is captured by the theoretical prediction, as shown in Fig. 7B.

## DISCUSSION

Here, the new experimental approach presented on the basis of a dielectrowetting setup allows the preparation of films in a well-defined initial configuration from which the subsequent dynamics of dewetting can be studied. It also allows the quantitative study of the evolution of the shape of a liquid as it dewets from an out-of-equilibrium film state to an equilibrium macroscopic droplet state. From one viewpoint, this can be interpreted as the reverse process of spreading of a droplet into film, in the sense that both the wetting and the dewetting proceed from an initial out-of-equilibrium state to a final equilibrium state through the interchange of solid-gas and solid-liquid interfaces. Our experiments and simulation results reveal that the full equilibration process of a circular dewetting liquid film into a single drop differs from the spreading counterpart. Upon release from an initial configuration that is far from equilibrium, the contact line recedes to relax the local curvature of the free surface. The emerging structure is a circular rim whose ends retract independently until they merge to form a spherical cap. Despite the smallness of inertial forces relative to viscous and capillary forces, the dewetting process is not a simple time reversal of the spreading problem, where a droplet undergoes a smooth sequence of spherical cap shapes as it relaxes from an out-of-equilibrium droplet shape toward a film configuration. This is because the mechanism driving the process stems from the local relaxation of the interface close to the contact line. Our simulations show that whether the initial film has an initial spherical cap shape or a slab shape, its initial dewetting is not a sequence of spherical cap shapes. The simulations also show that the dewetting behavior observed in our experiments is not specific to the liquid we have chosen, and so, our observations on the dewetting process have wide applicability.

The technique we have developed enables the study of the dynamics of dewetting liquids in detail and unlocks new opportunities for further quantitative experimental and theoretical studies of dewetting films. Possibilities include designing the configuration and applied voltage of the electrodes to create arbitrarily shaped films (for example, a ring or a star shape), which then dewet, and the combination of such shapes into arrays of dewetting droplets with simple or complex patterns of initial film shapes, including the creation of a dry patch within a film, which then proceeds to dewet (*13*). By considering different pairs of fluids or by adding surfactants, it is also possible to carry out experiments to study the effect of a viscosity contrast or surface tension in more detail. In the specific experiments we have presented, there is quantitative agreement with hydrodynamic theory, including previous predictions for film dewetting at intermediate times (*21*) and the final exponential relaxation of a spherical cap to equilibrium. Our experiments and lattice Boltzmann simulations allow us to identify the crossover time between both regimes, which is controlled by the initial aspect ratio of the film. This complete study fills a gap left by the previous inability to carry out direct experimental measurements of the film dynamics.

Here, we stress the significance of the slip length used in the theoretical model. In our experiments, where the typical molecular correlation length is on the order of nanometers, we expect the slip length to be of the same order. Fixing the slip length to a single value of λ = 1 nm for both the linear and exponential regimes leads to a quantitative prediction of the experimental data. Because the total viscous friction is an extensive property, its order of magnitude is determined by the length scale separation between the typical length scale of the dewetting rim, for example, its width *w*, to the molecular size λ. This is quantified by the weak logarithmic dependence on the ratio *w*/λ in Eq. 4, which sets the order of magnitude of the retraction speed for given values of the surface tension, viscosity, and equilibrium contact angle. For the macroscopic droplets used in our experiments, *w*/λ ~ 10^6^, Eq. 4 predicts a speed of retraction ~1 mm s^−1^, and thus, the typical rim dewetting time is τ_rim_ ~ 1 s. On the other hand, in our lattice Boltzmann simulations, we have fixed a comparatively larger slip length (see Materials and Methods), leading to a length scale separation where *w*/λ ~ 10. As a consequence, the typical retraction speeds are faster and the rim relaxation times are shorter. A signature of this effect is observed in the plateau for the contact angle observed in Figs. 2B and 4G, which is higher in the simulations than in the experiments. Using Eq. 5, we obtain the prediction θ/θ_e_ ≈ 0.84, in close agreement with our simulation results. Therefore, the slip length can be used to control the speed of retraction of the dewetting droplets. This result gives new insights into the role of interfacial slip in contact line dynamics and a new technique for studying interfacial slip. We hope that this will inspire further studies, where the effect of contact line slip on the retraction of the rim is investigated, for example, by using structured substrates, such as superhydrophobic (*24*) or liquid-infused surfaces (*25*), or by using complex fluids, such as colloid-polymer mixtures (*26*).

We close our discussion by pointing out the relevance of our results in other systems, where strongly distorted droplets undergo a dewetting process and where tuning of the solid-liquid interactions can lead to better control of the retraction speed of a liquid film. Our experiments and numerical simulations show that the emergence of a dewetting rim occurs when the initial aspect ratio of a liquid film is far from the equilibrium aspect ratio of the corresponding droplet shape. This condition can be quantified in terms of the initial height-to-radius ratio of the droplet and the equilibrium contact angle, giving *h*_0_/*R*_0_ << (1 − cosθ_e_)/sinθ_e_. These flattened films are not particular to drying situations but can arise in many situations involving fluid manipulation, such as spraying (*27*), drop impact (*28*–*30*), evaporation and condensation (*31*–*33*), convection (*34*), nucleate boiling (*35*), spin coating (*36*), and wetting on flexible surfaces (*37*–*39*).

## MATERIALS AND METHODS

### Experimental design

#### Electrode manufacture

**Fig. 8:**
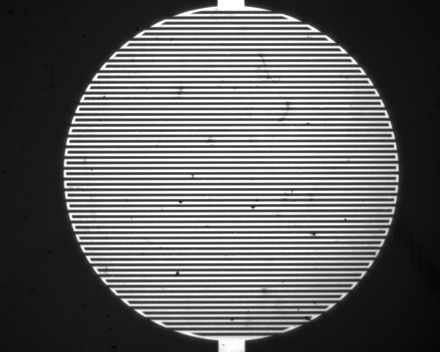
Teflon-coated IDEs. A circular electrode patch is defined from two interdigitated coplanar metal (Ti/Au) stripe arrays. The electrodes are covered by an insulating SU8 layer (1 μm), which is overcoated with a thin oleophobic layer of Teflon. The resulting pattern has a circular envelope 5 mm in diameter.

IDEs were produced by using a lift-off photolithographic method with a titanium-gold-titanium metallic layer. Glass slides (25 mm × 25 mm) were cleaned using 5, 0.5, and 0% solutions of Decon 90 detergent (Decon Laboratories) mixed with deionized (DI) water and ultrasonicated for 480 s and thoroughly rinsed with DI water between steps. The cleaned substrates were then soaked in 2-propanol (Fisher Scientific) and dried under nitrogen flux. The substrates were coated with S1813 G2 photoresist (Dow) to a thickness of 1.5 μm before soft baking at 110°C for 75 s. A SUSS MBJ4 mask aligner (Microtec) was then used to expose the photoresist-coated substrate with the 5-mm-diameter, 40-μm-linewidth/gap pattern over a circular area defined by the electrode lengths (Fig. 8). Exposed substrates were then developed using Microposit developer concentrate (Dow) mixed in a 50:50 solution with DI water to reveal the patterned substrate. Samples were metallized using a K575X sputter coater (Emitech) with a 10-nm gold film sandwiched between 5-nm layers of titanium. The photoresist was then stripped away using acetone (Fisher Scientific) to leave the patterned device. IDEs were cleaned again and coated with a 1-μm layer of SU8-2 (Dow), which acts as a dielectric layer and prevents electrical conduction through the liquid.

#### Electrical addressing, surface treatment, and temperature control

The electrical addressing to the IDEs was performed by a TGA1244 (TTI) arbitrary waveform generator providing 250-Hz sine wave to a PZD700 (Trek Inc.) amplifier, which gives an output of 420 V_r.m.s_. The uniformity of the output waveform was monitored using a TDS2014 oscilloscope (Tektronix Inc.), and the applied voltage was monitored using a 34410A digital voltmeter (Agilent). To encourage the retraction of the liquid, a hydrophobic coating of Teflon AF (Sigma-Aldrich) (θ_e_ ≈ 70°) was mixed in solution of 0.5% by weight and coated before baking at 60°C for 10 min to cure. Liquid droplets (1 to 2 μl) were dispensed onto the surface using a Microman pipette (Gilson). The IDE and liquid were then placed inside a Thermotron 3200 environmental chamber (Thermotron Industries), which was set to the required temperature and allowed to equilibrate for one hour before experimentation.

#### Image capture and analysis

Images were captured from the side and top of the liquid droplet using a DCC1545M-GL camera (Thorlabs) fitted with a 4× objective lens at 100 frames per second (FPS) and from the side using an HHC ×4 camera (Mega Speed Corporation) fitted with a 5× objective lens at 2000 FPS. To determine the contact angle and radius of the drop in the initial regime, a MATLAB program was developed to analyze the extracted frames using a user-determined baseline. *N*_p_ points above the baseline were used to fit a third-order polynomial, and this polynomial was then extrapolated to find the intersection with the baseline, which was used to calculate the radius of the droplet. The mean polynomial gradient at the intersect point was then used to calculate the angle between the polynomial and the baseline.

#### Lattice Boltzmann simulations

Simulations were carried out using the lattice Boltzmann algorithm detailed in the study by Desplat *et al*. (*18*). The simulation domain consists of a square lattice composed of “solid” and “fluid” nodes joined by links along the first and second nearest neighboring directions. At any given node of position vector **r**, we consider two sets of particle probability distribution functions, *f*_i_ and *g*_i_. The discrete time evolution in the lattice, with unit time step, is given by the single relaxation time lattice Boltzmann equations *f*_*i*_(**r** + **c**_*i*_, *t* + 1) = *f*_*i*_(**r**, *t*) − (*f*_*i*_ − *f*_*i*_^eq^)/τ_*f*_ and *g*_*i*_(**r** + **c**_*i*_, *t* + 1) = *g*_*i*_(**r**, *t*) − (*g*_*i*_ − *g*_*i*_^eq^)/τ_*g*_. Here, the dynamics includes a collision step, where the distribution functions relax toward equilibrium values, indicated by superscript “eq,” over the relaxation time scales τ_*f*_ and τ_*g*_. Subsequently, *f*_i_ and *g*_i_ are advected to neighboring nodes in a propagation step. The model considers a discrete set of advection velocities, {**c**_*i*_}, where the index *i* counts over the total number of advection directions, including rest particles. The hydrodynamic fields are defined through moments of the distribution functions, that is, ρ = Σ*f*_*i*_, ρ**u** = Σ**c**_*i*_*f*_*i*_ and φ = Σ*g*_*i*_, where ρ, **u**, and φ are the local density, velocity, and phase fields, respectively. Mass and momentum conservation is enforced by imposing the conditions Σ*f*_*i*_^eq^ = ρ, Σ*g*_*i*_^eq^ = φ, Σ**c**_*i*_*f*_*i*_^eq^ = ρ**u**, and Σ**c**_i_*g*_i_^eq^ = φ**u**. The thermodynamics of the fluid is specified through the equilibrium distribution functions. Here, we used a Cahn-Hilliard model to account for two immiscible fluids of arbitrary wetting angle 0 < θ_eq_ < π, with equilibrium phase-field values φ^eq^ = +1 and φ^eq^ = −1. In the long wavelength limit, the lattice Boltzmann algorithm is a good approximation to the hydrodynamic equations

$$\frac{\partial \rho }{\partial t}+u\cdot \nabla \rho =0$$ (10)

$$\frac{\partial \rho u}{\partial t}+(u\cdot \nabla )\rho u=-\nabla p+\mu \nabla^{2}u-\varphi \nabla \vartheta$$ (11)

$$\frac{\partial \varphi }{\partial t}+u\cdot \nabla \varphi =M\nabla^{2}\varphi$$ (12)

corresponding to the continuity, Navier-Stokes, and Cahn-Hilliard equations, where *p* and *ϑ* are the local pressure and chemical potential fields. The parameter mapping of the lattice Boltzmann algorithm to the hydrodynamic equations arises from the identification of the dynamic viscosity, μ, and phase-field mobility, *M*, with the relaxation time scales τ_f_ and τ_g_, respectively. In this formulation, contact line slip arises by virtue of diffusive transport close to the contact line, with a corresponding slip length determined by the fluid viscosity and phase-field mobility (*40*). In our lattice Boltzmann simulations, the slip length is comparable to the interface thickness, which is resolved over ~10 lattice sites. Because of computational constraints, the thickness of the dewetting film in our simulations is of order 10^2^ lattice sites. This leads to a scale separation between the film and interface thickness ~10. However, it has been recently shown that the slip length in the Cahn-Hilliard model can be mapped to the slip length used in sharp interface formulations in the context of the Cox-Voinov law for contact line dynamics over a wide range of length scale separations (*41*).

## Supplementary Material

http://advances.sciencemag.org/cgi/content/full/2/9/e1600183/DC1

## SUPPLEMENTARY MATERIALS

Supplementary material for this article is available at http://advances.sciencemag.org/cgi/content/full/2/9/e1600183/DC1 movie S1. Top view of a 1.45-μl thin liquid film dewetting from a Teflon-covered dielectrowetting 5-cm-wide circular patch at room temperature. movie S2. Side view of a 1.45-μl thin liquid film dewetting from a Teflon-covered dielectrowetting 5-cm-wide circular patch at room temperature.
