# Supplementary material for: Not spreading in reverse: The dewetting of a liquid film into a single drop
Source: Sci Adv. 2016 Sep 28;2(9):e1600183. doi: 10.1126/sciadv.1600183 (PMC5040479; doi:10.1126/sciadv.1600183)
Supplement: http://advances.sciencemag.org/cgi/content/full/2/9/e1600183/DC1 [file supp_2_9_e1600183__index.html]

Science Advances | Science Advances

## Supplementary Materials

**This PDF file includes:**

- Legends for movies S1 and S2

Download PDF

**Other Supplementary Material for this manuscript includes the following:**

- movie S1 (.avi format). Top view of a 1.45-μl thin liquid film dewetting from a Teflon-covered dielectrowetting 5-cm-wide circular patch at room temperature.
- movie S2 (.avi format). Side view of a 1.45-μl thin liquid film dewetting from a Teflon-covered dielectrowetting 5-cm-wide circular patch at room temperature.

**Files in this Data Supplement:**

- Adobe PDF - 1600183\_SM.pdf
