# Supplementary material for: Not spreading in reverse: The dewetting of a liquid film into a single drop
Source: Sci Adv. 2016 Sep 28;2(9):e1600183. doi: 10.1126/sciadv.1600183 (PMC5040479; doi:10.1126/sciadv.1600183)
Supplement: http://advances.sciencemag.org/cgi/content/full/2/9/e1600183/DC1 [file 1600183_SM.pdf]

Science Advances
AAAS
advances.sciencemag.org/cgi/content/full/2/9/e1600183/DC1

# Supplementary Materials for

## Not spreading in reverse: The dewetting of a liquid film into a single drop

Andrew M. J. Edwards, Rodrigo Ledesma-Aguilar, Michael I. Newton, Carl V. Brown, Glen McHale

Published 28 September 2016, *Sci. Adv.* **2**, e1600183 (2016)
DOI: 10.1126/sciadv.1600183

**The PDF file includes:**

- Legends for movies S1 and S2

**Other Supplementary Material for this manuscript includes the following:**
(available at advances.sciencemag.org/cgi/content/full/2/9/e1600183/DC1)

- movie S1 (.avi format). Top view of a 1.45-μl thin liquid film dewetting from a Teflon-covered dielectrowetting 5-cm-wide circular patch at room temperature.
- movie S2 (.avi format). Side view of a 1.45-μl thin liquid film dewetting from a Teflon-covered dielectrowetting 5-cm-wide circular patch at room temperature.

**Supplementary Materials**

movie S1. Top view of a 1.45-μl thin liquid film dewetting from a Teflon-covered dielectroewetting 5-cm-wide circular patch at room temperature.

movie S2. Side view of a 1.45-μl thin liquid film dewetting from a Teflon-covered dielectroewetting 5-cm-wide circular patch at room temperature.
